# Coronavirus-Induced Host Cubic Membranes and Lipid-Related Antiviral Therapies: A Focus on Bioactive Plasmalogens

**DOI:** 10.3389/fcell.2021.630242

**Published:** 2021-03-12

**Authors:** Yuru Deng, Angelina Angelova

**Affiliations:** ^1^Wenzhou Institute, University of Chinese Academy of Sciences, Wenzhou, China; ^2^Université Paris-Saclay, CNRS, Institut Galien Paris-Saclay UMR 8612, Châtenay-Malabry, France

**Keywords:** plasmalogen, cubic membrane, coronavirus, virus-host interaction, TEM, COVID-19

## Abstract

Coronaviruses have lipid envelopes required for their activity. The fact that coronavirus infection provokes the formation of cubic membranes (CM) (denoted also as convoluted membranes) in host cells has not been rationalized in the development of antiviral therapies yet. In this context, the role of bioactive plasmalogens (vinyl ether glycerophospholipids) is not completely understood. These lipid species display a propensity for non-lamellar phase formation, facilitating membrane fusion, and modulate the activity of membrane-bound proteins such as enzymes and receptors. At the organism level, plasmalogen deficiency is associated with cardiometabolic disorders including obesity and type 2 diabetes in humans. A straight link is perceived with the susceptibility of such patients to SARS-CoV-2 (severe acute respiratory syndrome-coronavirus-2) infection, the severity of illness, and the related difficulty in treatment. Based on correlations between the coronavirus-induced modifications of lipid metabolism in host cells, plasmalogen deficiency in the lung surfactant of COVID-19 patients, and the alterations of lipid membrane structural organization and composition including the induction of CM, we emphasize the key role of plasmalogens in the coronavirus (SARS-CoV-2, SARS-CoV, or MERS-CoV) entry and replication in host cells. Considering that plasmalogen-enriched lung surfactant formulations may improve the respiratory process in severe infected individuals, plasmalogens can be suggested as an anti-viral prophylactic, a lipid biomarker in SARS-CoV and SARS-CoV-2 infections, and a potential anti-viral therapeutic component of lung surfactant development for COVID-19 patients.

## Introduction

**FIGURE 1:**
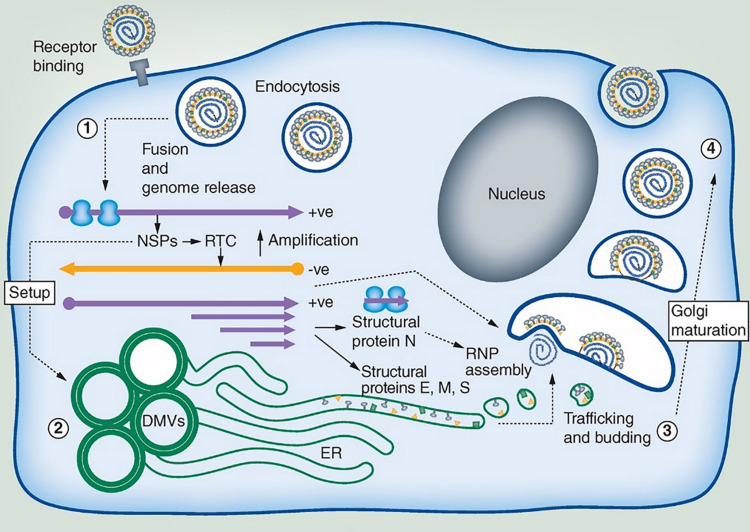
Coronavirus replication cycle highlighting areas where membrane interaction takes place (ER, Endoplasmic reticulum; DMV, Double-membrane vesicles; ERGIC, Endoplasmic reticulum Golgi intermediate compartments). SARS-CoV-2 viral particles consist of four proteins: S (“Spike”), M (“Membrane”), E (“Envelope”), and N (“Nucleocapsid”). The pathway of membrane interactions involves: (1) Viral internalization through binding of the viral spike (S) protein to the membrane protein receptor as human angiotensin-converting enzyme 2 (ACE2). The coronavirus particle enters the host cell by receptor-mediated endocytosis followed by RNA release and translation into virus polyproteins, which encode for non-structural proteins (NSPs). (2) NSPs stimulate the production of DMV compartments and the formation of replication transcription complexes (RTC). Translation of the structural proteins (M, E, and S) occurs in the ER membrane organelles. (3) Coronavirus assembly occurs in the intermediate compartment between the ER and ERGIC. The protein cargos migrate through Golgi stacks resulting in new virus particles that are embedded in vesicles (4). These vesicles can further fuse with the plasma membrane and egress. Reprinted from Alsaadi and Jones (2019) with permission.

All seven coronaviruses capable of infecting humans, including severe acute respiratory syndrome-coronavirus-2 (SARS-CoV-2), severe acute respiratory syndrome coronavirus (SARS-CoV), Middle East respiratory syndrome coronavirus (MERS-CoV), human coronavirus OC43 (HCoV-OC43), human coronavirus 229E (HCoV-229E), human coronavirus HKU1 (HCoV-HKU1), and human coronavirus NL63 (HCoV-NL63), employ lipid-binding domains for viral entry into host cells, intracellular lipid membrane modifications and host lipid reservoirs for viral replication and proliferation (Knoops et al., 2008; Miller and Krijnse-Locker, 2008; Ulasli et al., 2010; Oudshoorn et al., 2017; Abu-Farha et al., 2020). Lipids play an essential role during viral infection involving membrane fusion of virus to host cell, viral internalization through receptor-mediated or lipid-microdomain-mediated endocytosis, viral replication and viral exocytosis (Heaton and Randall, 2011; Belouzard et al., 2012; Li, 2016; Alsaadi and Jones, 2019). Figure 1 presents an earlier scheme about the viral entry and the replication cycle during coronaviruses infection. An emphasis is given on the locations of the membrane interactions, namely the endoplasmic reticulum (ER) from where double-membrane vesicles (DMV) and endoplasmic reticulum Golgi intermediate compartments (ERGIC) are generated. SARS-CoV-2 virus also enters the targeted host cell via endocytosis and fusion of the viral membrane with the host cell membrane.

Apart from endocytosis, coronaviruses such as SARS-CoV-2 can infect cells through direct fusion with the plasma membrane after activation of the human transmembrane protease serine 2 (TMPRSS2) (Li, 2015; Lukassen et al., 2020; Wan et al., 2020). The TMPRSS2 protein is essential for the viral infectivity by facilitating virus-cell membrane fusion through ACE2. The membrane fusion process between viral and host cells is a crucial step during coronavirus infection (Goldsmith et al., 2004; Wu et al., 2012). After binding of the surface-exposed spike protein trimer of SARS-CoV-2 virus to its high affinity receptor angiotensin-converting enzyme 2 (ACE2) in the host membranes, the viral entry occurs through fusion of glycoprotein and remodeling of host cell membranes (Li et al., 2003, 2005; Li, 2015; Wan et al., 2020). The membrane fusion has been considered to be several fold more efficient than endocytosis during viral infection. The role of lipid membrane properties in this process have not been examined in details. Membrane fusion of SARS-CoV and of SARS-CoV-2 results in RNA release into the cytoplasm of host cell followed by viral replication (Alsaadi and Jones, 2019).

The (+)ssRNA viruses exploit diverse intracellular membranes in host cells in order to assemble RNA replication complexes (Stapleford and Miller, 2010; Heaton and Randall, 2011) through creating compartments for viral genome amplification. Lipids from host cells are used for generation of lipid membrane envelope shape called double-membrane vesicles (DMV) (Figure 1). Coronavirus-induced DMVs are formed by protrusion and budding of the endoplasmic reticulum (ER) cisternae followed by the detachment of closed vesicular objects (Oudshoorn et al., 2017; Zhang et al., 2020). The organelle-like membranous replicative structures serve as sites of viral RNA synthesis. Of interest, they are generated through topological transitions and curvature changes of the ER membranes. DMVs have been observed upon remodeling of convoluted membranes or reticulovesicular networks occurring after deformation and compartmentization of the continuous ER membranes (Knoops et al., 2008; Oudshoorn et al., 2017; Snijder et al., 2020; Zhang et al., 2020).

During SARS-CoV-2 virus life, the S-protein adopts closed and open conformations (Yan et al., 2020). High resolution structural studies of the receptor-binding domains (RBD) of coronavirus S proteins have established that they involve pockets of a tube-like shape and a size, which matches that of a free fatty acid molecule when in a closed conformation of S protein (Yan et al., 2020). Several studies have supported the evidence that lipid-binding domains in virus proteins are essential for virus replication (Stapleford and Miller, 2010; Jeon and Lee, 2017; Yan et al., 2020). Moreover, it has been demonstrated that viral proliferation requires the increased fatty acid and cholesterol biosynthesis as well as release of free fatty acids from lipid droplets (Su et al., 2002; Miller and Krijnse-Locker, 2008; Heaton et al., 2010; Heaton and Randall, 2010; Yin et al., 2010; Jeon and Lee, 2017). An exogenous supply of linoleic acid (LA) and arachidonic acid (AA) has been shown to inhibit the viral replication of MERS-CoV and HCoV-229E (Yan et al., 2019). LA and AA are polyunsaturated omega-6 fatty acids, which modulate the activity of enzymes including membrane receptor proteins and ion channels of the host cells (Das, 2018; Das, 2020a,b,c,d, 2021).

A free-fatty-acid-binding pocket in the locked structure of SARS-CoV-2 spike protein has been revealed by cryo-EM analysis (Yan et al., 2020). The cryo-EM image of SARS-CoV-2 spike (S) glycoprotein has indicated that the receptor binding domains entrap linoleic acid (LA) in composite binding pockets present also in the revealed 3D structures of SARS-CoV and MERS-CoV coronaviruses. It has been emphasized that free-fatty-acid binding pocket resembles a bent tube, which well fits the size and shape of linoleic acid (LA) (Yan et al., 2020). LA is a metabolic precursor of AA, which mediates host defensive inflammatory response. The ability of SARS-CoV-2 to sequester LA in the binding pockets has been suggested as a tissue-independent mechanism in coronavirus infection, which leads to host inflammation process. Tight LA binding can stabilize the locked conformation of the S-protein in SARS-CoV-2 coronavirus, which may lead to diminished interaction with host ACE2 receptor.

Coronavirus infection modifies both lipid composition and membrane structure, topology and trafficking of the host cells in order to ensure virus particle replication and proliferation. Thus, host lipid biogenesis is crucial for the viral life cycle and replication. Host cell lipid alterations upon coronavirus infection have been analyzed by ultra-high-performance liquid chromatography (UPLC) and mass spectrometry (MS)-based lipidomics approach (Yan et al., 2019). It has been affirmed that viral infection re-programs host lipid metabolism for the purposes of viral proliferation (Schoggins and Randall, 2013). Glycerophospholipids and fatty acids (FAs) have been found to be significantly upregulated in HCoV-229E-infected host cells. HCoV-229E viral infection has increased the levels of lyso-phospholipids [lysoPCs (16:0/0:0) and lysoPEs (16:0/0:0)] and also unsaturated/saturated FAs arachidonic acid (AA), linoleic acid (LA), palmitic acid (PA), and oleic acid (OA) (Yan et al., 2019). For coronaviruses, the LA-AA metabolic pathway is indispensable for host lipid remodeling (Zaman et al., 2010) and has been highlighted as a niche for therapeutic interventions (Yan et al., 2020).

**FIGURE 2:**
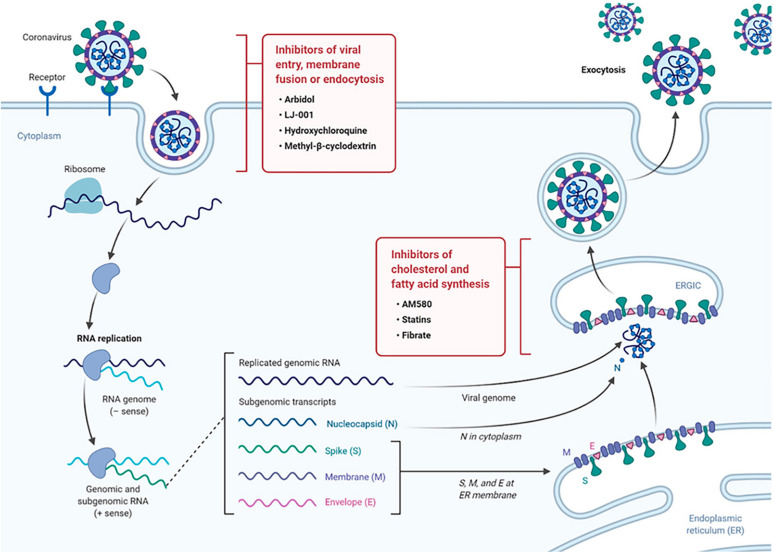
Drug targeting strategies in viral infection exploiting the role of lipid metabolism. The scheme of the life cycle of SARS-COV-2 indicates the locations where lipid-modifying drugs may act as broad-spectrum antiviral compounds to inhibit viral entry, membrane fusion, endocytosis or host cholesterol and fatty acid biosynthesis. Reprinted from Abu-Farha et al. (2020) with permission.

The possibilities of targeting host lipid metabolism and host membrane trafficking in order to inhibit the viral cycle have been intensely discussed recently (Abu-Farha et al., 2020; Das, 2020a,b,c,d, 2021; Glebov, 2020; Xiu et al., 2020). Such approach is much less susceptible to the development of viral resistance as compared to the strategies focusing on viral mutations. Targeting lipid metabolism thus has been suggested as an alternative antiviral strategy (Das, 2020a,b,c,d, 2021). Figure 2 presents the advances in antiviral drug development including (i) inhibition of fatty acid and cholesterol synthesis, and (ii) inhibition of viral entry, membrane fusion, or endocytosis.

Of interest, cholesterol is involved in multiple steps of the coronavirus life cycle, and therefore targeting cholesterol has been suggested as an antiviral strategy (Abu-Farha et al., 2020). Cholesterol is well distributed in the microdomains of cell membranes. Considering that microdomains are implicated in coronavirus-host membrane interactions, drugs that alter microdomain formation and composition have been tested in antiviral approaches. An example of a drug with antiviral activity, which targets lipid metabolism, is statin. It is well known that statin impairs the biosynthetic pathway of cholesterol. As an inhibitor of cholesterol synthesis, statin has been considered as a generic drug against SARS-CoV-2 and other related viruses, among other agents that are specific for inhibition of fatty acid biosynthesis (Figure 2).

The development of therapeutic compounds that target the cell membrane has stimulated the emergent field of membrane lipid therapy (MLT) (Escribá et al., 2015). The new therapeutic approach aims at regulation of the lipids in pathological biomembranes (Escribá, 2017). Thus, the cellular membranes, rather than specific proteins alone, constitute the therapeutic targets. The rationale of this strategy accounts for the fact that coronaviruses cause extensive host cell membrane perturbations. Therefore, host membrane rearrangements have been considered as a target as a novel remedy for antiviral drug development.

An example of a drug for coronavirus membrane targeting is 2-hydroxyoleic acid (Minerval^®^). Minerval interacts with the membrane lipids and modifies the composition and structure of host cellular membrane (Prades et al., 2008). The drug influences the phospholipid packing in the polar region of lipid bilayers at the interface with contact proteins and increases the non-lamellar propensity of the lipid membrane assembly. The resulting increased bilayer fluidity may permit (i) deeper hydrophobic regions of the membrane to interact with hydrophobic domains of peripheral proteins, or (ii) fatty acid moieties of phospholipids to protrude out of the bilayer plane. These structural effects can activate anchored enzymes such as PKC and sphingomyelin synthase involved in the lipid metabolism process.

Although detergents and detergent-based disinfectants may permeabilize the lipid membrane shell of coronavirus by creating pores and thus destroy the virion, they cannot be used as antiviral drugs in humans because they have the risks of causing death of the patients due to the massive destruction of cell membranes. At variance, antiviral lipids may alter the membrane properties and trafficking as well as affect signaling and intracellular transport dynamics, which should be explored as antiviral strategies.

**FIGURE 3:**
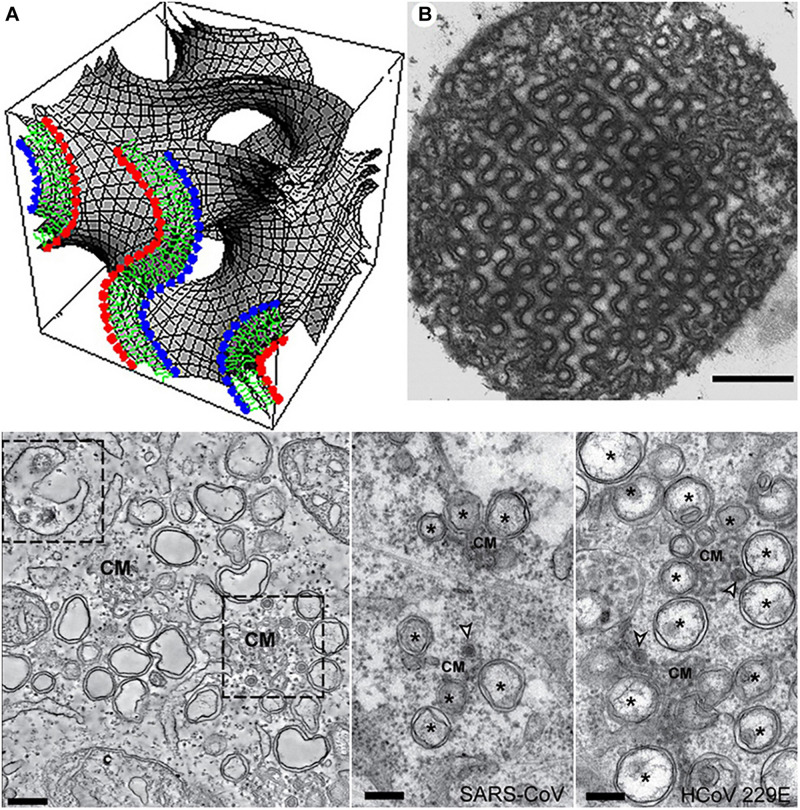
Top panel: Cubic membrane topology represented by a mathematical model of the lipid bilayer organized on a 3D cubic lattice **(A)** and 2D-projected transmission electron microscopy (TEM) image of cubic membranes found in the mitochondria of 10-day starved ameba Chaos cells **(B)**. Scale bar: 500 nm. Reprinted from Deng and Almsherqi (2015) with permission. Bottom panel: TEM images of interconnected convoluted membrane structures (CM) induced by MERS-CoV infection in mammal Huh7 cells (left) and SARS-CoV and HCoV-229E coronavirus induced convoluted membranes (CM), double-membrane vesicles (DMV), and double-membrane spherules (middle and right). Scale bars: 250 nm. Reprinted from Snijder et al. (2020) with permission.

Membrane folding and formation of three-dimensional (3D) lipid membrane topologies can be provoked by changes in the membrane lipid composition, protein clustering, bilayer bending caused by embedded or anchored proteins, or lipid membrane curvature alteration under the influence of environmental factors (Almsherqi et al., 2006). Whereas lipids of a cylinder-like shape (like the typical glycerophospholipids) favor the formation of lamellar structures, lipids of a truncated-cone molecular shape tend to form curved membrane assemblies such as bicontinuous cubic, bicontinuous sponge or inverted hexagonal structures (Angelova et al., 2021). Among them, cubic membranes are characterized by periodic structural arrangement of bicontinuous lipid bilayers organized in cubic lattice networks (Figure 3, top panel).

Bilayer lipid membranes may rearrange into 3D cubic membranes under stress conditions, which correspond to either altered lipid metabolism or protein overexpression (Almsherqi et al., 2006) in disease states as well as other types of environmental stress including viral infection (Deng et al., 2010), pH changes, presence of ions or solutes of increased concentrations, temperature changes, light, electric field, *etc*. (Almsherqi et al., 2009). Out-of-plane membrane shape deformations of interconnected bilayers have been termed convoluted membranes (Knoops et al., 2008; Oudshoorn et al., 2017; Snijder et al., 2020; Figure 3, bottom panel). Formation of cellular cubic membranes or convoluted membranes can be induced through reprogrammed lipid metabolism, altered lipid-protein interactions or by specific protein-protein interactions. Such non-lamellar structures are considered as transient states associated with the membrane bending, instabilities and rearrangement caused by the non-lamellar phase transition.

## Open Questions in the Current Focus

Accumulating evidence suggests that lipid treatment of virus-infected cells is a strategy for SARS-CoV-2 inhibition (Das, 2020a,b,c,d, 2021). Whereas the role of different phospholipid and fatty acid types has received prior attention in the pathology of cardiometabolic disorders, obesity, and type 2 diabetes, the question regarding how to exploit the individual lipid classes against coronavirus SARS-CoV-2 entry and replication is still at an initial stage of comprehension. Arachidonic acid and other polyunsaturated fatty acids, as well as their derived metabolites, have been proposed to serve as antiviral molecules to inactivate the enveloped viruses and inhibit their proliferation (Das, 2020a,b,c,d, 2021). However, are there other antiviral lipid species to offer the options for the treatment?

**FIGURE 4:**
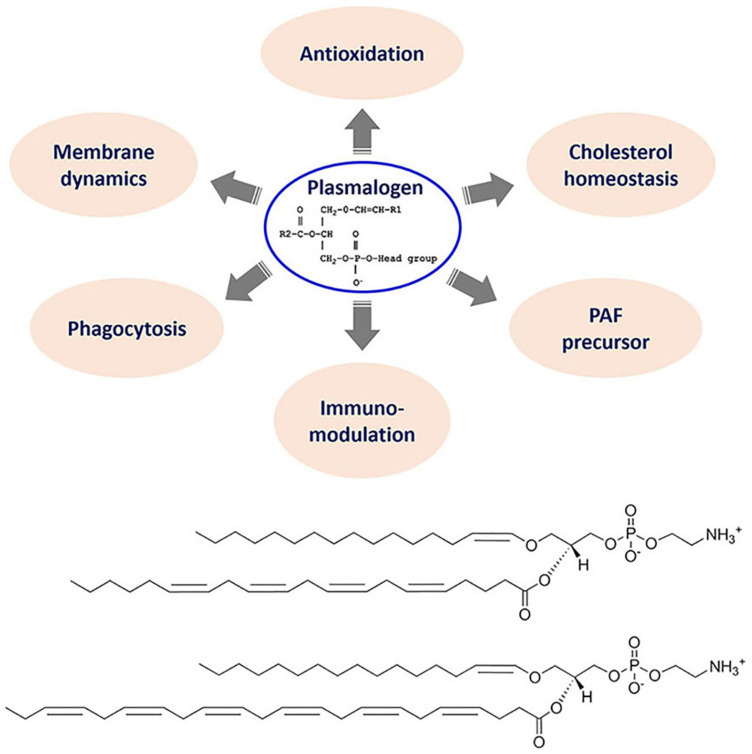
Top panel: General chemical structure and multiple biological functions of bioactive plasmalogens. Bottom panel: Chemical structures of exemplary ether phospholipids (plasmalogens) involving arachidonic acid (AA) and docosahexaenoic acid (DHA) chains, *e.g.*, 1-(1Z-hexadecenyl)-2-arachidonoyl-sn-glycero-3- phosphoethanolamine [C16(Plasm)-20:4 PE] and 1-(1Z-hexadecenyl)-2-docosahexaenoyl-sn-glycero-3-phosphoethanolamine [C16(Plasm)-22:6 PE].

Here we focus on the special phospholipid class of “plasmalogen,” the bioactive molecules as antiviral prophylactics and potential constituents of combination treatments against COVID-19. Plasmalogens are special ether phospholipids that are critical for cell membrane integrity in terms of structure and function. In addition to their role as key membrane components, they are involved in the regulation of cholesterol homeostasis and macrophage phagocytosis in addition to immunomodulation (Figure 4). Moreover, their antioxidant properties may determine the outcome of host illness during viral infections. Biosynthesis of endogenous plasmalogens requires functional peroxisomes, the oxidative cell organelles in almost all the eukaryotes. The deficiency of plasmalogens implicated in cardiometabolic and multiple neurodegenerative diseases may render humans host susceptible to SARS-CoV-2 (COVID-19) and other similar viral infections (SARS-CoV or MERS-CoV).

The plasmalogen lipid type has received less attention in the comprehension of the coronavirus infection and therapy as compared to free fatty acids like linoleic acid (LA) and arachidonic acid (AA) (Das, 2020c; Yan et al., 2020). It is of current interest to explore how plasmalogens may participate in the stages of virus-host interaction process including: (1) viral entry host cells via non-receptor microdomain-mediated endocytosis pathways; (2) lipid-modulated host innate immune response; (3) virus-induced host membrane rearrangements, especially cubic membrane (CM) formation. Plasmalogen deficiency, due to impaired nutrition, stress and contemporary lifestyles, does not support host CM formation during viral infections. It will be emphasized here that induced host CM architectures may serve as an evolutionally conserved antioxidant defense system to favor viral proliferation in a controlled manner and also the fast return of host homeostasis.

Another question is whether a host response treatment approach to viral infections may essentially reduce the severity of COVID-19 illness and improve patient survival. In fact, the balanced immune and inflammatory response is the key for host to live and not to die after SARS-CoV-2 infection. The shift of virus-targeted therapies to host response treatment, especially lipid and membrane factors, might be an alternative solution for the host survival. The presented concept here is based on our previous opinion paper “Do viruses subvert cholesterol homeostasis to induce host cubic membranes?” (Deng et al., 2010). We further ask the questions: (i) What is the role of plasmalogens in the remodeling of host lipid membrane? and (ii) What would be the role of plasmalogens in antiviral therapy? Moreover, the potential applications of plasmalogen-preconditioning treatment and prophylaxis are discussed.

## The Significance of Plasmalogen Lipid Class

Plasmalogens constitute a class of ether phospholipids containing a fatty alcohol with a vinyl-ether bond at sn-1 position and a polyunsaturated fatty acid at sn-2 position of the glycerol backbone (Figure 4). Compared to their diacyl counterparts, plasmalogens appear to be highly fusogenic lipids (Glaser and Gross, 1995) enriched in lipid microdomains (Pike et al., 2002) and tend to form more densely packed and thicker bilayer membranes (Rog and Koivuniemi, 2016). Plasmalogens are significant structural components of various subcellular organelle membranes including nucleus, endoplasmic reticulum (ER), post-Golgi network and mitochondria (Honsho et al., 2008). Plasmalogens are determinants in membrane dynamics and trafficking. A recent structural study has revealed that they can strongly influence the membrane thickness and curvature (Angelova et al., 2021). They also serve as endogenous antioxidants, protect against ROS, and prevent lipoprotein oxidation.

Plasmalogens are abundant in human brain, heart, kidney, lung, skeletal muscle and immune cells (Braverman and Moser, 2012). Lipidomic profiling of multiple human cohorts have identified negative associations between plasmalogens and obesity (Weir et al., 2013), type 2 diabetes (Meikle et al., 2013) and cardiovascular diseases (Meikle et al., 2011), supporting the concept of the vital role of plasmalogens in the prevention of cardiometabolic diseases (Paul et al., 2019).

Plasmalogens are prominent for regulation of cholesterol homeostasis (Honsho et al., 2015), which has previously attracted great attentions as a key host factor during multiple viral infections (Deng et al., 2010). Having both enriched in lipid microdomains (Pike et al., 2002), the existence of a metabolic relationship between cholesterol and plasmalogen is therefore directly emergent. Fedson et al. (2020) proposed the prophylaxis use of statins (generic drug to lower cholesterol) for the previous epidemic and current pandemic including influenza (Fedson, 2006), Ebola (Fedson, 2016) and COVID-19 (Fedson et al., 2020). Statin drugs display anti-inflammatory and immunomodulatory effects in the host response treatment approach. Despite that the potential use of plasmalogens in achieving cholesterol homeostasis has been proposed as an alternative to statin therapy (Mankidy et al., 2010), further studies are required toward the translation of plasmalogens into future clinical applications.

Plasmalogens, by virtue of their vinyl ether bond and enrichment in PUFAs moieties (AA and DHA chains), play vital roles in many cellular processes. They provide unique membrane structural attributes to potentially modulate lipid-associated signaling pathways and protecting important macromolecules (nucleic acids and lipids) from oxidative damages (Almsherqi et al., 2008; Deng and Almsherqi (2015). Their metabolic products of DHA and AA derived from physiological breakdown of plasmalogens via phospholipase A2 (PLA2) have been shown to increase the expression of inflammatory cytokines, which also increase the activity of plasmalogen-specific PLA2 (Farooqui, 2010). The neuroinflammatory modulation of plasmalogens has been reported in a couple of studies (Ifuku et al., 2012; Katafuchi et al., 2012).

## Viral Entry Depends on Host Membrane Lipid Compositions: A Focus on Plasmalogens

Viral infection initiates with the virus particles crossing the host plasma membrane, often via receptor-mediated endocytosis pathway (Figure 1). However, an alternative non-receptor lipid-microdomain-mediated endocytosis and membrane fusion process may proceed as well. The non-specific lipid-mediated viral entry may possibly explain the puzzling zoonotic viral transmission via jumps of pathogenic viruses between different species (*e.g.*, from bat to human). Even though virions mainly enter host cells through specific proteinaceous receptors, such as ACE2/TMPRSS2 in the case of SARS-CoV-2, the strengthened or weakened attachment and entry of viruses depend on the lipid composition of viral envelopes in addition to host plasma membranes. Although plasmalogens similar to cholesterols have been found to be enriched in membrane microdomains (Pike et al., 2002), the scarce plasmalogen research somehow impedes our understandings of this special lipid class and the potentially significant roles they may act in virus-host interactions.

The increased levels of plasmalogens have been detected in the serum of ZIKA infected subjects (Queiroz et al., 2019) and chronic HBV patients (Schoeman et al., 2016). The strong enrichment of plasmalogens has been noticed in virion lipidome of human cytomegalovirus (Liu et al., 2011) and HIV (Brugger et al., 2006). Moreover, the virus-induced alterations in both plasmalogen levels and peroxisome activity have been examined at host level (Satoh et al., 1990; Farooqui and Horrocks, 2001; Dixit et al., 2010; Odendall and Kagan, 2013; Martin-Acebes et al., 2014).

The endogenous biosynthesis of plasmalogens requires functional peroxisomes in almost all the eukaryotes. Peroxisomes are the important host organelles where certain viral replication may take place (Cook et al., 2019). Peroxisome plasticity in virus-host interaction and its role as double-edged sword in multiple viruses’ infections have attracted great attention recently. The increased levels of plasmalogens in the serum of ZIKA (flavivirus) viral infected subjects suggested a strong link between ZIKA virus life cycle and host peroxisomes. The observation that flaviviruses induce peroxisome-mediated lipid alterations in the host cells may further explain the established upregulation of plasmalogens in the serum of ZIKV infected patients (Martín-Acebes et al., 2019; Queiroz et al., 2019). Meanwhile, plasmalogen PC was also reported to play a pivotal role in influenza virus infection (Tanner et al., 2014).

## Coronavirus-Induced Host Cubic Membrane Formation for Both Virus and Host Fitness

**FIGURE 5:**
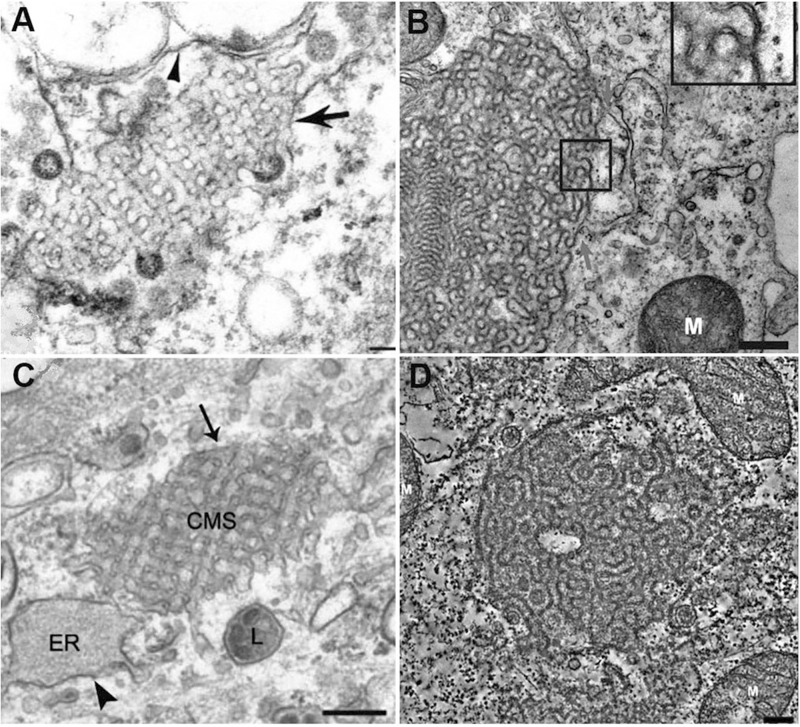
Multiple examples of coronavirus-induced cubic membrane (CM) formation in the host cells. **(A)** SARS-CoV, 3d post-infection (p.i.) Vero-E6 cell with virus particles egress (Goldsmith et al., 2004) **(B)** MERS-CoV nsp3-6, 24h post-transfection, Huh-7 cell (Oudshoorn et al., 2017). **(C)** MHV-59, 8h p.i. HeLa-CEACAM1a cell (Ulasli et al., 2010); **(D)** SARS-CoV (nsp3 + nsp4), 24h post-transfection, 293T cell (Oudshoorn et al., 2017).

Severe acute respiratory syndrome-coronavirus infection induces multiple alterations of the lipid membrane architecture in the host cells. Remodeling of endoplasmic reticulum (ER) cisternae upon coronavirus infection starts with detachment of closed vesicular objects called double-membrane vesicles (DMV) (Alsaadi and Jones, 2019; Zhang et al., 2020). In addition to the generation of DMV lipid envelopes (referred to as coronavirus replication organelles), the host membrane may adopt 3D topologies called “convoluted membranes” (Knoops et al., 2008; Zhang et al., 2020). They form through fusion of multiple DMV or other type of structural transitions (Knoops et al., 2008). Examples of such host membrane rearrangements are illustrated by the transmission electron microscopy (TEM) images in Figure 5.

In the absence of a clear appreciation of the 3D nature of cytomembraneous inclusions observed in the TEM micrographs of virus-induced host membrane complexes, various terminologies have been used to denote the formation of 3D non-lamellar assemblies of folded membranes (Almsherqi et al., 2005). For instance, “tubule-crystalline inclusions” have been described in HCV-infected liver, “convoluted membraneous mass” in viral St. Louis Encephalitis, and “tubule-reticular structures (TRS)” in AIDS as well as in multiple coronavirus infections including SARS-CoV infected Vero cells (Goldsmith et al., 2004; Almsherqi et al., 2005). TRS has been considered as a specific ultrastructural marker of AIDS in various organs (Maturi and Font, 1996). Most of the time, the 3D membrane rearrangements, observed also in MERS-CoA infected Huh-7 cells (Oudshoorn et al., 2017) and coronavirus MHV infected Hela cells (Ulasli et al., 2010; Figure 5) have been termed “Convoluted Membranes” (Knoops et al., 2008; Oudshoorn et al., 2017; Snijder et al., 2020; Zhang et al., 2020). They have been unified by the name “Cubic Membranes” in the last decade (Deng et al., 2010; Deng and Almsherqi, 2015).

Using the direct template matching method in electron microscopy, we have characterized TEM micrographs of host membrane rearrangements, induced by multiple viral infections, as Cubic Membranes (CM) (Almsherqi et al., 2006; Deng et al., 2010). Although the molecular mechanisms behind viral-induced host CM remains unclear, CM was proposed to act as antioxidant defense system in ameba *Chaos* cell model (Deng and Almsherqi, 2015; Deng et al., 2017). This concept fairly supports our current hypothesis that virus-induced CM may not only benefit for viral proliferations in terms of viral assembly and egress (Figure 5A), but CM may also help the return of host homeostasis by mitigating the oxidative damages (Deng and Almsherqi, 2015) during viral infection to further promote cell survival (Chong et al., 2018).

In addition to the antioxidant properties of CM nanostructures, plasmalogens pre-conditioning treatment or supplementation may determine the degree of host CM abundance during viral infections. This hypothesis is based on our findings from ameba cell model system (Chong et al., 2018). We have previously asked an intriguing question whether viruses subvert cholesterol homeostasis to induce host CM (Deng et al., 2010). At that time, we were not aware of the emergent links of plasmalogens in multiple viruses’ life cycle and the corresponding host CM formation. At present, we highlight the close relationship between plasmalogen and cholesterol metabolism as both species are enriched in membrane rafts microdomains. These microdomains and their associated lipid molecular types might have been overlooked in the process of viral entry and host immune signaling. Plasmalogen pre-conditioning is indispensable in ameba CM formation under cell starvation stress conditions, and it may also apply to host stress response during coronavirus infections. Therefore, the role of CM in the virus-host interaction and balance, host cell response and survival appear of significance.

## Peroxisome-Mediated Antiviral Immune Signaling: A Focus on Plasmalogens

The host response to infection may turn out to be the key determinant of pathogenesis of emergent infectious diseases including the current COVID-19 pandemics. The first step of virus life cycle is to enter the host cells by crossing the plasma membrane, where the lipid composition might be very crucial. Upon viral infections in humans, peroxisomes act as vital immune signaling organelles, aiding the host by orchestrating antiviral signaling (Cook et al., 2019). Peroxisomes are critical host organelles emerging as a double-edged sword during the progression of viral infections. These cellular organelles, that both host or kill pathogen, can make use of their functions to achieve host antiviral defense or to be hijacked to serve for viral proliferation (Karnati and Baumgart-Vogt, 2008).

The peroxisome was first recognized as a key subcellular signaling center upon the discovery of mitochondrial antiviral signaling (MAVS), an innate host immune response. MAVS, previously thought to be exclusive to the mitochondria, mounts up a rapid antiviral reaction. However, combined with the known detoxification functions of peroxisomes, the recognition of peroxisomal MAVS has resulted in the realization of peroxisomal role in host defense as an antiviral signaling organelle. This was supported by the studies of human cytomegalovirus (HCMV) and herpes simplex type 1 (HSV-1) infections, along with the discovery that these viruses promotes host peroxisome biogenesis during infections (Beltran et al., 2018). Certain cellular lipids, including very-long-chain fatty acids (e.g., DHA) and ether lipids (e.g., plasmalogens), can only be synthesized in peroxisomes.

Plasmalogens, the peroxisome-synthesized lipids are intriguing candidates for various viral infection-induced cell processes, including the construction of viral envelopes, modulation of host cholesterol homeostasis, and maintenance of virus-host balance and fitness. With the knowledge that plasmalogens are enriched in HCMV virions, Liu et al. (2011) have proposed that peroxisomal lipid metabolism might be a general feature of enveloped virus infections. In support of this, one study of influenza virus, another RNA virus, showed that host ether lipid metabolism was required and enhanced upon infection, and that peroxisome-derived plasmalogens were enriched in influenza virions (Tanner et al., 2014). Plasmalogen lipids are the key component of several enveloped viruses, including HCMV (Liu et al., 2011), WNV (Martin-Acebes et al., 2014), and influenza. We thus highly suspect that this may also apply to SARS-CoV-2. In this perspective, further lipidomic analysis of COVID-19 samples will be required for a more detailed picture.

## The Role of Plasmalogens in Host Immune Response During Viral Infection: A Focus on Macrophages

Macrophages, as the professional phagocytes of host immune system, are capable of detecting and clearing invading pathogens (e.g., viruses) and damaged cells through phagocytosis. Macrophages are essential in host innate immunity and tissue homeostasis in addition to inflammatory modulation and response. Plasmalogen deficiency in macrophages was associated with their reduced phagocytosis, and this reduction was significantly reversed when the cells were exposed to lyso-plasmalogen PE (Rubio et al., 2018). In parallel, restoration of plasmalogen level in macrophages also increased the number and size of lipid microdomains in the membranes of macrophages. The exogenous administration of plasmalogens was thus considered as a potential strategy to optimize the functions of macrophages.

A study on human monocyte to macrophage differentiation, performed by a bioinformatic approach combined with transcriptomic and lipidomic analyses (Wallner et al., 2014), has demonstrated that plasmalogen PE is a potential biomarker of immune system activation. The authors have further pointed out that the dysregulation of monocyte-macrophage differentiation is a hallmark of vascular and metabolic diseases and associated with persistent low grade of inflammation. In parallel, diminished plasmalogen levels have been observed in the obese subjects (Wallner et al., 2014). All these findings have brought our deep interest to the potential link of host plasmalogen dysregulation and high morbidity and mortality of COVID-19 patients.

Another related ether lipid, such as platelet-activating factor (PAF), may participate in the severe pathology of COVID-19 pneumonia as well. PAF was the first intact phospholipid known to have messenger functions and a mediator of inflammation in the mechanism of human immune response (review see Lordan et al., 2019). PAF is powerful for the activation of human inflammatory cells at extremely low concentrations, which imparts a hormone-like character. PAF is present in many cell types, especially those involved in host defense such as platelets, endothelial cells, neutrophils, monocytes and macrophages. PAF has evolved as a part of protective mechanism in host innate defense system, and with a number of pro-inflammatory properties necessary for host protection from pathogenic insults. When produced in an uncontrolled manner, PAF may have extremely harmful effect, including blood clotting problem which has been reported in severe COVID-19 illness. The relation of PAF precursors to plasmalogen metabolites is indicated in Figure 4 as a part of the ether lipid role overview.

The increased plasmalogen content may induce the formation of lipid rafts microdomains and further improve the recruitment, oligomerization, and interaction of receptors and signaling proteins involved in the phagocytosis of macrophages. Plasmalogens also reduce the non-lamellar-to-lamellar phase transition temperature, exhibiting a high propensity to form non-lamellar phase structures (Lohner et al., 1991). The non-lamellar structural intermediates are indeed essential for membrane fusion process. The increased abundance of plasmalogens carrying the polyunsaturated fatty acid moieties in the serum of ZIKA patients may explain its important implication in viral particle fusion with host cell membranes. On the other side, polyunsaturated AA and DHA fatty acids carried by plasmalogens are the substrate sources for generation of soluble lipid mediators, which participate in host immune signaling and inflammatory responses.

## Potential Role of Plasmalogens in the Cytokine Storm Observed in COVID-19 Patients. Interplay With a Lipid Storm

Cytokines are a diverse group of small proteins that are secreted by cells for the purposes of intercellular signaling and communication. They include interferons (IFNs), interleukins (ILs), chemokines, and tumor necrosis factors (TNFs). The multiple functions of cytokines span from control of cell proliferation and differentiation to immune and inflammatory responses, which are highly relevant to developing viral infections (Barry et al., 2000; Imashuku, 2002; Yokota, 2003; Jahrling et al., 2004; Huang et al., 2005) such as SARS-CoV-2 (COVID-19) (Castelli et al., 2020; Leisman et al., 2020; Mulchandani et al., 2020).

The devastating “cytokine storms” occurs when the host immune homeostasis is broken due to viral infection and inflammatory responses flaring out of control. They are associated with a wide variety of infectious and non-infectious diseases and may even be an unfortunate consequence of therapeutic intervention attempts. Increased inflammation and a cytokine storm characterize the COVID-19 cases by severe pneumonia that can decompensate to an acute respiratory distress syndrome (Castelli et al., 2020; Leisman et al., 2020; Mulchandani et al., 2020).

In addition to the recognized cytokine storm previously documented (Castelli et al., 2020; Leisman et al., 2020; Mulchandani et al., 2020), analyses of lung fluids of SARS-CoV-2-infected patients have indicated that a “lipid storm” also occurs. Using liquid chromatography combined with tandem mass spectrometry, a recent report has quantified several lung bioactive lipids and has evidenced that the “lipid storm” in severe SARS-CoV-2 infections involves both pro- and anti-inflammatory lipids (Archambault et al., 2020). Bronchoalveolar lavages of severe COVID-19 patients contained large amounts of the bioactive lipids prostaglandins (PGs), leukotrienes (LTs), and thromboxanes (TXs) (Archambault et al., 2020). The established increased oxidative bust in the lungs of severe COVID-19 patients has pointed out the importance of the lipid storm taking place in the lungs of pneumonia patients.

**FIGURE 6:**
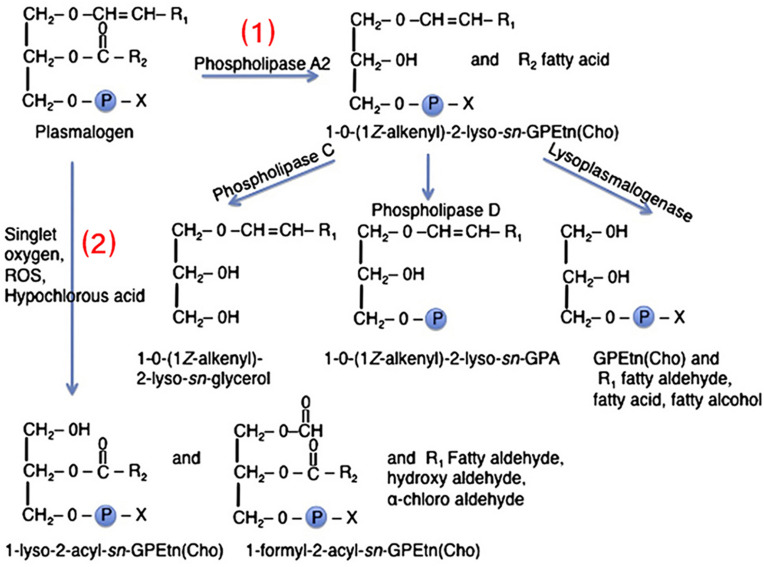
Two pathways of plasmalogen turnover, remodeling and degradation: (1) Through phospholipase A2 (PLA2) and (2) through oxidative stress. Plasmalogens are one of the primary targets of HOCl due to sensitivity of the vinyl-ether bonds to oxidation. X denotes the polar head group, which is typically ethanolamine or choline. R1 denotes the carbon chain at the sn-1 position, and R2 at the sn-2 position. Reprinted from Braverman and Moser (2012) with permission.

**FIGURE 7:**
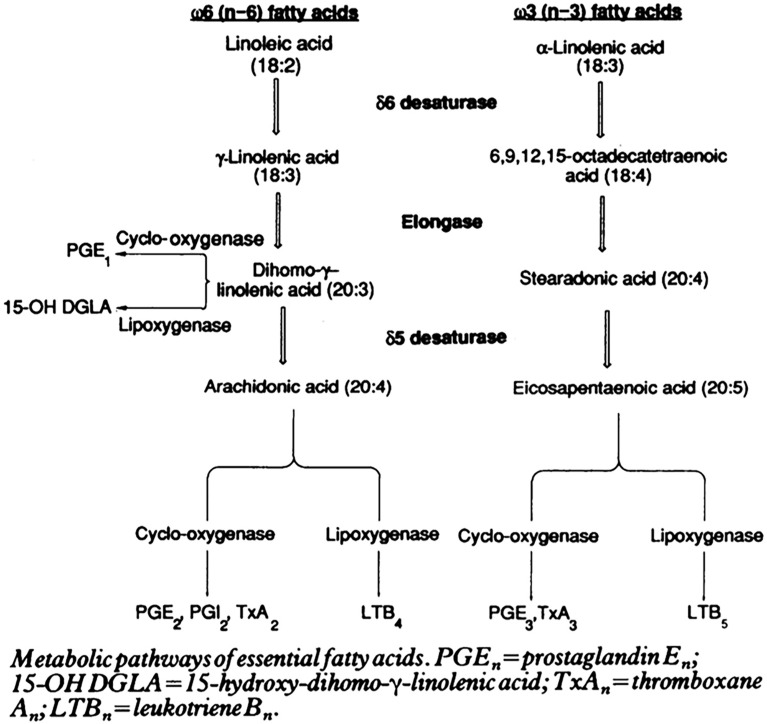
Essential fatty acids in inflammation and potential “lipid storm” in severe COVID-19 patients. Scheme of eicosanoid and related bioactive lipid mediators production due to metabolic pathways of fatty acid alteration (eicosanoid related precursors). Reprinted from Zurrier (1991) with permission.

The role of plasmalogens in the cytokine and lipid storms remains to be experimentally elucidated. Figures 6, 7 summarize the pathways of plasmalogen turnover, remodeling and degradation, which help identifying the metabolites and the products that may trigger uncontrolled inflammatory responses.

Plasmalogens often carry PUFAs, which can be either of the omega-6 (pro-inflammatory) or of the omega-3 (anti-inflammatory) families (e.g., AA or EPA and DHA). Both types can be catalyzed by cyclooxygenase (PGs, TXs) and lipoxygenase (LTs) in the production of eicosanoids [prostaglandins (PGs), thromboxanes (TXs), and leukotrienes (LTs)] (Figure 7). The balance between these two families is important for the host immune homeostasis, which determines the potential development of undesired lipid storm. The controlled formation of eicosanoids is regarded as beneficial because it may help optimize cellular defensive reactions against the invading pathogens including SARS-CoV-2. However, excessive, uncontrolled production of eicosanoids is associated with the “lipid storm” (Archambault et al., 2020). Eicosanoid signaling is a pro-inflammatory component of innate immunity as the cytokine signaling. Unfortunately, lipid storm can be self-destructive in interplay together with the peptide-mediated cytokine storm. Devastating consequence may emerge in the lungs and spread to other tissues in the body of severe COVID-19 patients.

Plasmalogens, enriched in leukocytes, are one of the primary targets of hypochlorous acid (HOCl) due to the sensitivity of the vinyl ether bond to oxidative agents (Figure 6; Üllena et al., 2010; Braverman and Moser, 2012). The ether lipids, in contrast to their counterpart ester phospholipids, are the targets of HOCl generated by leukocyte myeloperoxidase as a part of the immune defense reaction (respiratory bust). The direct products, α-chloro fatty aldehyde and lysophospholipids, may produce a family of chlorinated lipids that can regulate inflammatory responses. Further inflammatory cascades may deplete the plasmalogen levels (Braverman and Moser, 2012).

In addition to this breakdown due to oxidative stress, plasmalogens can be hydrolyzed by phospholipase A2 (PLA2) into products like lyso-pPE and PUFAs (AA, EPA, or DHA) (Üllena et al., 2010). The lysophosphatidylethanolamine plasmalogen (lyso-pPE) has been identified as a self antigen for natural killer T cells (NKT cells). It is important for the development, the maturation, and the activation of iNKT cells in the thymus, which is vital for innate immunity (Facciotti et al., 2012; Ni et al., 2014). The lyso-pPE is very potent at low nanomolar concentrations and may induce cytokine release from freshly isolated iNKT cells. While this activity is important in immunomodulation, which has been considered as a sensor of inflammation, the proper stimulation of iNKT cells is significant for the protection against autoimmunity (Van Kaer et al., 2013).

To our knowledge, there are no reports on how PUFA metabolism (PGs, LTs, TXs) can be altered by plasmalogens in order to clarify their impact on cytokines. The essential PUFAs metabolism and its role in inflammation have been reviewed by Das (2019) with a special focus on eicosanoids [prostaglandins (PGs), leukotrienes (LTs), and thromboxanes (TXs)]. Eicosanoids and docosanoids are important signaling molecules produced by the oxidation of omega-6 arachidonic acid (AA) or other omega-3 PUFAs, eicosapentaenoic acid (EPA) and docosapentaenoic acid (DHA) from the cell membrane phospholipid pool. Das (2021) has drawn attention to the fact that pro-inflammatory metabolites like prostaglandin E2 (PGE2) and leukotrienes (LTs) (derived from AA) and anti-inflammatory lipoxin A4 (LXA4) as well as resolvins, protectins, and maresins (derived from EPA and DHA) facilitate the generation of M1 (pro-inflammatory) and M2 (anti-inflammatory) macrophages, respectively. Moreover, AA, PGE2, and LXA4 among others inhibit the synthesis of interleukin-6 (IL-6) and tumor necrosis factor-α (TNF-α) (Das, 2019, 2021).

**FIGURE 8:**
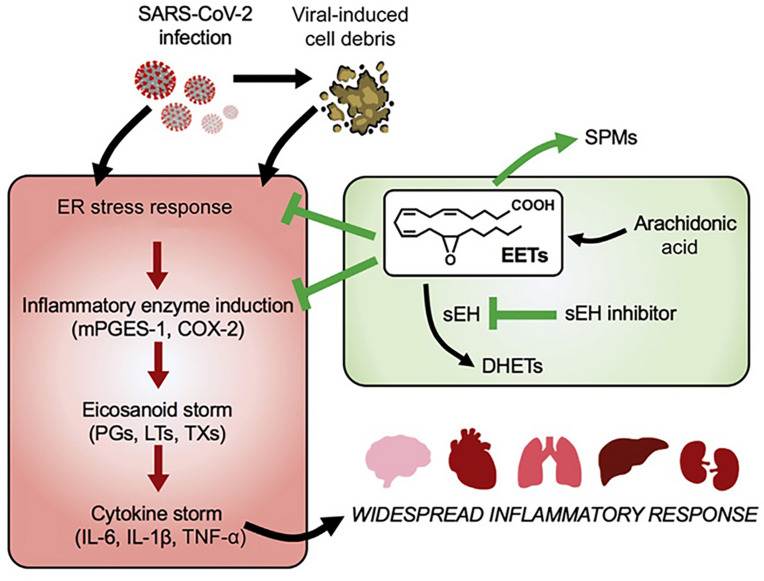
Lipid (eicosanoid) storm may occur before the cytokine storm in SARS-CoV-2 infection. Eicosanoids are bioactive lipid mediators derived from oxygenated polyunsaturated fatty acids (PUFAs). Reprinted from Hammock et al. (2020) with permission.

Despite that eicosanoid and cytokine storms are not well characterized in coronavirus infection yet, published works indicate that lipid (eicosanoid and related bioactive lipid mediators) storm might occur before the cytokine storm (Figure 8). Coronavirus SARS-CoV-2 infection triggers endoplasmic reticulum (ER) stress response, which may be followed by subsequent eicosanoid and cytokine storms (Hammock et al., 2020). Targeting of the proinflammatory eicosanoids, including PGs, LTs, TXs, would be beneficial for diminishment of the ER stress. With the contribution of lipidomics, a better understanding of the eicosanoid storm, preceding the cytokine storm in severe inflammation (Figure 8), should provide insights toward new strategies for management of coronavirus infection.

**FIGURE 9:**
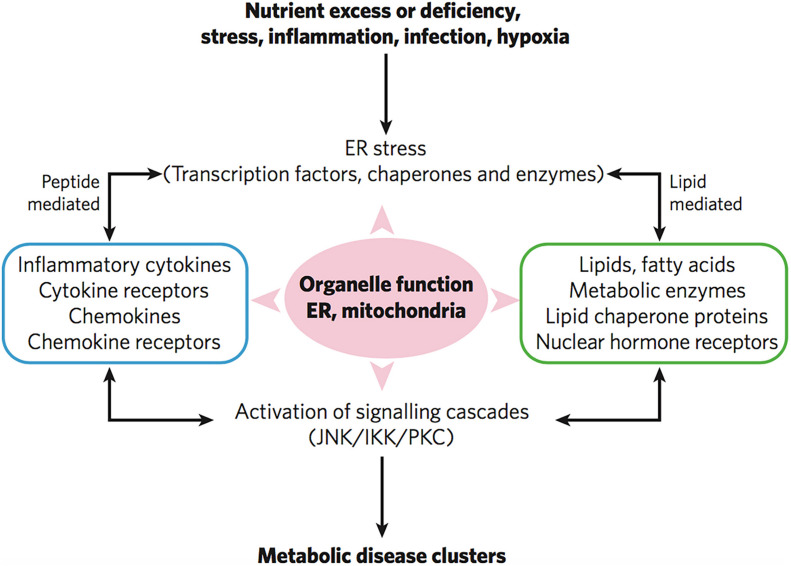
Severe infections trigger inflammatory responses, ER stress and mitochondrial organelle dysfunction through lipid-mediated and cytokine peptide-mediated mechanisms. Reprinted from Hotamisligil (2006) with permission.

Taken together, coronavirus infection is associated with both inflammation and metabolic disorders. Such disorders cause durable damage of subcellular organelles including mitochondrial defects and endoplasmic reticulum (ER) dysfunction (Hotamisligil, 2006). Mitochondrial defects and ER dysfunction are crucial in the activation of various inflammatory pathways and widespread inflammatory responses (Figure 8). Figure 9 represents the impact of the lipid-mediated and cytokine-mediated metabolic and inflammatory cascades at the organelle level. In a further step, this impact should be considered at the lipid membrane level, where the plasmalogen lipid type plays an important role. Plasmalogens yielding docosanoids [bioactive oxygenated polyunsaturated fatty acids (22:6n-3) containing 22 carbons] with anti-inflammatory functions might be the key lipid components helping to inhibit the inflammation.

Regarding the potential role of plasmalogens in cytokine storm seen in COVID-19, we speculate that the host immune homeostasis is broken in response to coronavirus infections. However, with the sufficient level of membrane plasmalogens, it may support the host viral-induced CM formation (Deng et al., 2010). CM formation can lower the oxidative damage (Deng and Almsherqi, 2015; Deng et al., 2017) and promote the quick return of host immune homeostasis, otherwise the host may have higher risk of developing the uncontrolled lipid storm and further entangled with cytokine storms.

## Plasmalogens in Lung Surfactant and Antiviral Pulmonary Surfactant Therapies

Coronavirus infection impairs the capacity of the type 2 alveolar epithelium cells to synthesize and secrete pulmonary surfactant required for normal breathing and oxygenation (Schousboe et al., 2020; Zheng et al., 2020). In healthy individuals, lung surfactant covers the alveolar surface, facilitates breathing, and prevents the lungs from collapsing (Hallman et al., 1982; Daniels and Orgeig, 2003). Dysfunctional endogenous lung surfactant in the patients with severe respiratory pathologies provoked by SARS-CoV-2, may be due to the decreased concentrations of surfactant phospholipid and proteins, the altered lung surfactant metabolism and composition, or oxidative inactivation of surfactant proteins (Ahmed et al., 2020; Schousboe et al., 2020). Considering that pulmonary surfactant synthesis is diminished by the severe acute respiratory syndrome-coronavirus-2 pathology, potential therapies of COVID-19 caused pneumonia may include exogenous lung surfactant replacement or delivery to compensate the deficiency of surfactant lipids, which are strongly associated with lung pathogenesis (Mirastschijski et al., 2020).

There is a close interaction and crosstalk between lung surfactant and phagocytosis behavior of alveolar macrophages (Juers et al., 1976; Spragg et al., 2004; Diler et al., 2014; Tschernig et al., 2016). Interestingly, the alveolar macrophages do not kill *in vitro* the pathogens unless the latter have been incubated with alveolar lining material (i.e., lung surfactant) before their phagocytosis. Although plasmalogens in lung surfactant are not the major components of the phospholipid mixture, they have been reported to significantly reduce surface viscosity and surface tension (Rüdiger et al., 1998, 2005; Zhuo et al., 2021). The role of plasmalogens in facilitating membrane fusion has been well recognized (Marrink and Mark, 2004). In this regard, studies with fibroblasts derived from plasmalogen-deficient human patients have shown marked inhibition of exocytosis and endocytosis processes (Perichon et al., 1998; Thai et al., 2001).

**FIGURE 10:**
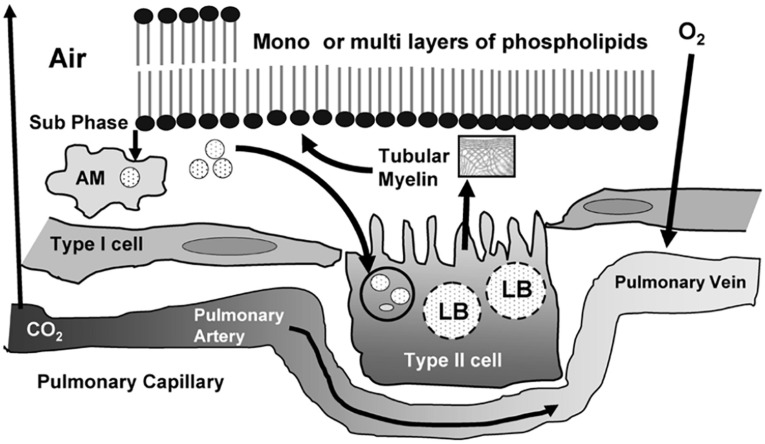
Life cycle of surfactant produced in the lung with an indication of the tubular myelin, lamellar bodies (LB), and alveolar macrophages (AM). Pulmonary surfactant is a surface-active lipo-protein complex produced by type II alveolar cells. Reprinted from Ramanathan (2006) with permission.

Lung surfactant is a multicomponent mixture of lipids (phospholipids, triglycerides, fatty acids, and cholesterols), surfactant proteins, and a small amount of carbohydrates (Bernhard, 2016; Schousboe et al., 2020). The lipid components comprise saturated and unsaturated phospholipids, neutral lipids, and ether lipids (plasmalogens). Phosphatidylcholine (PC) is the predominant lipid class accounting for about 50% of the phospholipids of lung surfactant. Phosphatidylethanolamine (PE) accounts for up to 20% of phospholipids, whereas phosphatidylserine (PS), phosphatidylinositol (PI), and phosphatidylglycerol (PG) constitute 12–15% of the total phospholipid content of lung surfactant. The antiviral activity of the lung surfactant might be due to the pulmonary lipids that may inhibit virus-mediated host inflammation and infection. Of interest, surfactant-associated proteins are required for the formation of tubular myelin (described by a deformed P-based cubic membrane surface) (Larsson and Larsson, 2014) which promotes the adsorption of lipid molecules at the air/water interface and play a role in the monolayer film stability (Figure 10).

Plasmalogen is the minor trace but critical component of lung surfactant. This ether phospholipid, together with cholesterol and surfactant-associated proteins, regulate the surfactant monolayer stability and viscosity. Another key feature is that plasmalogens act as antioxidants and protect alveolar cells from oxidative stress that often encountered at alveolar surface (Zhuo et al., 2021). Plasmalogens may contribute to substantial lowering the surface tension (Rüdiger et al., 1998) and viscosity (Rüdiger et al., 2005) of the lipid mixture. It has been emphasized that lower surfactant viscosity may enhance the clinical response to the therapy of respiratory pathology (Bernhard, 2016).

**FIGURE 11:**
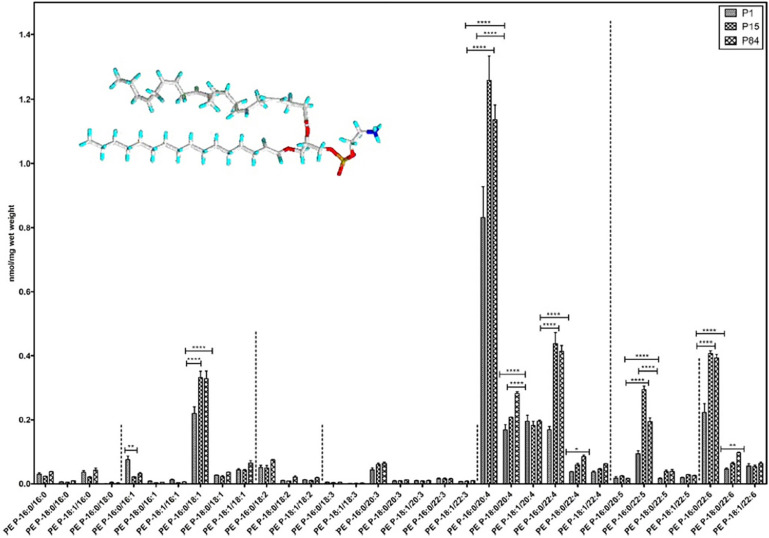
Composition of individual phosphatidylethanolamine PE-based plasmalogen (PE-P) lipid species during postnatal development of mouse lung. High contents of 20:4, 22:6, 22:5, and 22:4 plasmalogen (PE-P) derivatives are detected. PE P-16:0 (sn-1) plasmalogens are present in higher amounts in all 4 groups of ethanolamine plasmalogens, whereas PE P-18:0 and PE P-18:1 account for smaller amounts. Values are represented as nmol/mg wet weight. Reprinted from Karnati et al. (2018) with permission. Statistical significance (*^,^ **^,^ ***): *p*-values <0.05 (significant), < 0.01, < 0.001 (highly significant).

Quantitative lipidomic analysis of mouse lung during postnatal development, using electrospray ionization tandem mass spectrometry, has determined the individual plasmalogen lipid types in the pulmonary surfactant (Karnati et al., 2018). Phosphatidylethanolamine (PE)-based plasmalogens have been found to be much more abundant as compared to ether-phosphatidylcholines (PC) during the postnatal mouse lung development. Figure 11 shows that PE-based plasmalogens comprise a high content of 20:4, 22:6, 22:5, and 22:4 chains likely to be arachidonic acid (AA), docosahexaenoic acid (DHA), docosapentaenoic acid (DPA), and adrenic acid-rich plasmalogen derivatives. Evidently, this lipid content determines the capacity of the lung surfactant to protect the alveolar epithelium cells from hypoxia and ROS-mediated stress and to reduce the risk of respiratory distress diseases.

**FIGURE 12:**
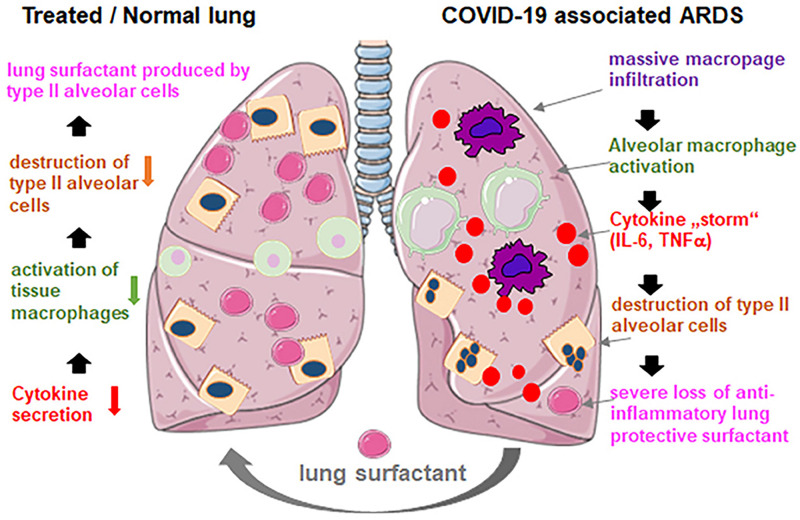
Exogenous lung surfactant delivery suggested as a therapy to reduce inflammation and restore pulmonary barrier in severe COVID-19 associated acute respiratory distress syndrome (ARDS). Reprinted from Mirastschijski et al. (2020) with permission.

Recently, it has been claimed that the lung injury caused by SARS-CoV-2 coronavirus in the pulmonary tissue of COVID-19 pneumonia patients developing acute respiratory distress syndrome (ARDS) strongly resembles the effects of neonatal respiratory distress syndrome (NRDS) (Schousboe et al., 2020). Both disorders are associated with lung surfactant deficiency (Mirastschijski et al., 2020). The clinical consequences from the impact of SARS-CoV-2 on the alveolar type II cells, and the production and turnover of pulmonary surfactant, are associated with alveolar collapse and inflammation, which leads to increased capillary permeability, edema, and microvascular thrombosis (Figure 12). The viral infection may cause alterations in the whole lung lipid composition. Moreover, vascular permeability increases as the pulmonary pathology progresses and the pulmonary surfactant gets deactivated, which makes the lungs unstable with time (Walmrath et al., 1996).

It has been proposed that early administration of natural lung surfactant may improve the pulmonary function in COVID-19 patients with severe ARDS (Mirastschijski et al., 2020). The suggested pulmonary surfactant therapy (Walther et al., 2019; Ahmed et al., 2020; Mirastschijski et al., 2020; Pramod et al., 2020) is motivated by the fact that lung surfactant administration is a safe and efficient therapy for neonates with ARDS (Walmrath et al., 1996). Therefore, pulmonary surfactant therapy envisions the development of lung surfactant formulations for pulmonary barrier restoration in patients with COVID-19 pneumonia (Walther et al., 2019; Mirastschijski et al., 2020; Pramod et al., 2020). It is expected that this approach may improve the treatment outcome of COVID-19 patients.

Whereas the levels of plasmalogens might be insufficient for COVID-19 patient treatment in the clinical studies employing natural lung surfactants, we propose plasmalogen-enriched lung surfactant formulations as the next generation therapeutic opportunity toward more efficient pulmonary surfactant therapy against COVID-19 pneumonia. Taking into account the results of the lipidomics analysis about the major ether lipid components (Karnati et al., 2018), it appears urgent to develop the synthetic surfactants enriched in phosphatidylethanolamine (PE)-based plasmalogens as those presented in Figure 11.

## Inclusion of Plasmalogens in Combination Therapies Against Coronavirus Infections

We hypothesize that the inclusion of plasmalogens in antiviral formulations for combination therapy of severe COVID-19 pneumonia patients may enhance the overall efficacy of anti-SARS-CoV-2 treatment. This antiviral lipid class may render the treatment resistant to eventual viral protein mutations during the viral propagation of the pathology. Both membrane lipid therapy and pulmonary surfactant therapy with plasmalogen-enriched antiviral formulations should be applicable to most of the coronaviruses including SARS-CoV-2, SARS-CoV, and MERS-CoV.

In the absence of clinical data for severe COVID-19 patients, it is early to argue whether plasmalogens have a role simply because they contain PUFAs. Current understanding highlights that the PUFAs content of plasmalogens is important in prevention and management of COVID-19. Das (2021) has emphasized that the PUFA bioactive lipid arachidonic acid (AA, 20:4n-6) has a capacity to inactivate SARS-CoV-2, facilitate macrophage generation, suppress inflammation, and prevent vascular endothelial cell damage, which opens new perspectives for therapeutic uses of AA in anti-coronavirus strategies.

Ongoing early stage inhibitory therapies involve the design of potential entry inhibitors against SARS-CoV-2. Strategies for blocking the viral entry consider that the virus utilizes the angiotensin-converting enzyme 2 (ACE2) as an entry receptor in human cells. Therefore, the S protein is an important target for the development of anti-SARS-CoV-2 therapeutics. Blocking the binding of S protein to ACE2 can be done either by fatty acids or by peptides and antibodies, which will inhibit the SARS-CoV-2 virus proliferation. Peptide therapeutics are promising antagonists in this regard (Struck et al., 2012). However, considering the molecular diversity of the coronavirus entry receptors of host cells (Millet et al., 2020), this targeting mechanism might not be sufficient to stop the pandemics. The analysis of COVID-19 lung samples has revealed a dramatic upregulation of the interferon gamma (IFNγ) protein, which may be accompanied by a large innate immune response (Hu et al., 2020).

Various potential therapeutic approaches (Imai et al., 2005; Struck et al., 2012; Monteil et al., 2020), some of which are highlighted in Figure 2, can be combined in multi-therapies toward the aim of SARS-CoV-2 inhibition:

- Development of spike protein-based vaccines;
- Antibody therapy using antibody molecules for targeting and neutralization of the spike proteins, which mediate the viral entry;
- Delivering excessive quantity of soluble form of ACE2;
- Blocking ACE2 receptor;
- Inhibition of transmembrane protease serine 2 (TMPRSS2) activity;
- Using antivirals (RNA polymerase inhibitors) to stop the viral replication;
- Using short interfering RNAs directly or indirectly targeting the viral DNA replication machinery;
- Drugs targeting lipid metabolism;
- Exogenous interferon gamma (IFNγ) delivery to compensate its deficiency in susceptible to virus infection.

In analogy with the human immunodeficiency virus (HIV) treatment regimen using tri-drug combinations, coronavirus research may envision future triple therapies or multi-therapies against SARS-CoV-2 coronavirus infection. Plasmalogens can be included in the category of membrane-lipid targeted therapies in approaches of treating the coronavirus and/or host membranes as antiviral targets. In combination therapies with other antiviral agents, plasmalogens will be significant as antiviral lipids and lung surfactant ingredients to treat severe COVID-19 pneumonia.

## Concluding Remarks

The current COVID-19 pandemics is similar to the previous severe acute respiratory syndrome (SARS-CoV, 2002–2003) and Middle East Respiratory Syndrome (MERS-CoV, 2012-ongoing) outbreaks. All these coronavirus infections are traced through zoonotic transmission. All have similar clinical manifestations mainly as lower respiratory tract diseases with significant mortality especially in the elderly with underlying chronic illnesses (obesity, type 2 diabetes and cardiometabolic diseases).

Plasmalogens are a class of membrane ether glycerophos pholipids with unique properties displaying a propensity for non-lamellar phase formation that may strongly influence the activity of membrane-bound enzymes and receptors. Plasmalogens are crucial in human health and disease and playing roles in immune signaling and as endogenous antioxidants. Increasing evidence supports the correlation between diminished levels of plasmalogens and a number of pathological states including neurodegenerative and cardiometabolic disorders as well as the severe acute respiratory distress syndrome due to the coronavirus infections. Dysregulated levels of plasmalogens are found in infected patients as a result of coronavirus-induced modification of the lipid metabolism. This strongly indicates that plasmalogen is among the key lipids potentially modulating the viral infection.

Based on the features discussed above, we suggest the potential role of plasmalogen pre-conditioning as anti-viral therapeutic and prophylaxis strategy. Along the line, plasmalogen pre-conditioning may promote host cubic membrane (CM) formation in response to multiple stress and diseased conditions including coronavirus infections. CM has been proposed as an evolutionary antioxidant defense system Deng and Almsherqi (2015). The antioxidant plasmalogen molecules participate in the host CM formation and CM in return to act as antioxidant defense system. Host cubic membrane induction in virus infected cells has not been rationalized in the development of antiviral therapies yet. However, the discussed multiple correlations and phenomena here lead to the conclusion that the plasmalogen lipid type is of great interest and significance for the future COVID-19 therapy and might be considered as a biomarker in SARS-CoV-2 infection and treatment. Further work in each of these areas will be necessary to realize the full potential of plasmalogen modulation and CM formation as therapeutic strategies in membrane lipid therapy and antiviral combination remedies for the next pandemics.

## Author Contributions

YD and AA conceived and wrote the manuscript. Both authors contributed to the article and approved the submitted version.

## Conflict of Interest

The authors declare that the research was conducted in the absence of any commercial or financial relationships that could be construed as a potential conflict of interest.
